# Relationship between craving to drugs, emotional manipulation and interoceptive awareness for social acceptance: the addictive perspective

**DOI:** 10.1186/s12912-023-01556-7

**Published:** 2023-10-11

**Authors:** Mahmoud Abdelwahab Khedr, Ayman Mohamed El-Ashry, Eman Abdeen Ali, Rasha Salah Eweida

**Affiliations:** 1 Psychiatric and Mental Health Nursing Department, Faculty of Nursing, Alexandria University, Alexandria, Egypt; 2 Medical Surgical Nursing Department, Faculty of Nursing, Alexandria University, Alexandria, Egypt

**Keywords:** Craving, Manipulation, Awareness, Acceptance, Substance use disorder

## Abstract

**Background:**

Drug addiction (DA) is a global psychiatric worldwide problem. Patients with substance use disorder are more likely to use the numerous defenses at their disposal to control their surroundings emotionally. This could virtually cause a tidal wave of social rejection of them in the community. The study aims to investigate drug craving, emotional manipulation, and interoceptive awareness for social acceptance among patients with substance use disorder.

**Methods:**

This study followed a descriptive correlational design on a sample of 110 patients with substance use disorder who were recruited to complete the Penn Alcohol Craving Scale, the Emotion Manipulation Questionnaire, and the Perceived Acceptance Scale.

**Results:**

Most respondents recorded high levels of PACS and emotional manipulation ability. A highly positive and significant correlation was found between scores on emotional manipulation ability and PACS.

**Conclusion:**

Craving for drugs was a significant predictor of emotional manipulation ability. Incorporation of effective nursing interventions to enable patients with substance use disorder to engage in self-reflection related to how their cravings for drugs may lead them to prioritize their needs over others.

## Background

Drug addiction (DA) is a global psychiatric problem affecting about 10% of people worldwide [1]. Drug craving is one of the most prevailing features associated with addictive disorders. Drug craving means “the self-conscious experience of desire to use drugs“ [2]. It is typically intruding on conscious awareness and dominating the thoughts of patients with substance use disorder (SUD), thereby evoking an augmented feeling of distress. The magnitude of distress pervades massive areas of their lives, including academics, work performance, and social relationships, and subsequently worsen their quality of life [3]. Besides, it could virtually cause a tidal wave of social rejection of them in the community. In that sense, shedding the light and providing a lens on such a looming problem.

It is persuasively argued that patients with substance use disorder are unable to withhold from the braying demands of addiction. They are more likely to exhibit a wide array of socially unacceptable behaviors that result from the dynamics of their illness [4]. One of the most prevalent behaviors is the tendency to emotionally manipulate others to meet their immediate needs and desires. Such kinds of covert behavior are commonly demonstrated without respecting the interlocutors’ needs or feelings [4]. Emotional manipulation means “the ability of persons to manipulate the emotions of others within a self-serving framework [5]. Park (2021) proclaimed that addictive manipulators are more subjected to using the myriad defenses that are available to them to control their surroundings’ emotionally [3].

## Theoretical background

At the theoretical level, manipulative behavior is based on the premise that individuals are compelled to perceive manipulators’ verbal messages uncritically. In this scenario, the manipulator tries to create illusions and misperceptions to target or affect the surroundings’ emotions to fulfill the beneficial actions of the manipulator [6]. The spectrum of emotional manipulation behavior encompasses two facets: positive and negative. On the other hand, patients with substance use disorder can express genuine sorrow or tears for what they do and seek sympathy and forgiveness. In addition, they could demonstrate flattery, gift-giving, and asking for special privileges or favors. The negative facet is that patients with substance use disorder manipulate others by evoking feelings of helplessness in their interlocutors. They frequently engage in evasion, lying, stealing, dishonesty, bargaining, and guilt-tripping [7]. Taken together, such socially unacceptable behaviors might prevent patients with substance use disorder from extracting maximum benefit from their social support system, including family members, friends, or loved ones [8].

Lack of social acceptance is one of the hallmarks of patients with substance use disorder [9]. Social acceptance means “the other people signal that they wish to include the individual in their groups and relationships [10]. From a social-psychological perspective, the consequent behavior of addiction fosters the erosion of patients’ social lives because of the displayed manipulative behaviors [11]. In this case, the patients’ families, friends, and other surroundings might feel emotionally abused by the patients with substance use disorder’s manipulative behavior. The factor that would propagate a host of negative emotions in the patients with substance use disorder’s social context, leading to their desires to expel themselves from the existing relationship. Emerging evidence has shown that caregivers of patients with substance use disorder often perceive their behaviors as overly demanding. Over time, they felt ashamed and bonded less with them. Mannelli (2013) reported that the manipulative behavior of patients with substance use disorder infiltrated the social lives of their surroundings and resulted in a missing sense of love, belonging, or even being empathized with [12].

## Significance of the study

According to World Health Organization (WHO) statistics, around 270 million individuals worldwide, aged between 15 and 64, are estimated to be affected by substance use disorders. This statistic indicates that the disorder affects approximately 5.5% of a given population [13]. More specifically, the National Addiction Research Study (2018) recorded the prevalence of substance use disorder is 33% in Cairo, 22.4% in Upper Egypt, and 9.6% in Delta [14]. Releasing from the scientific literature and building on voluminous prior studies, the relationship between craving for drugs, emotional manipulation, and interoceptive awareness for social acceptance in the psychiatric trajectory is still not wholly intelligible. For this reason, we called for this study to fill this gap in literature. Our study would provide a reference epitome to frame a potentially nuanced relationship among the interesting variables. This, by virtue, allows psychiatric nurses to become more comprehensive and adopt proficient nursing interventions designed to assist patients with substance use disorders. These maneuvers would enable them to introspect inside themselves to gain more self-awareness about how intensifying their desire for drugs could push them to pursue their own needs at the expense of others.

## Aim of the study

This study aims to investigate drug craving, emotional manipulation, and interoceptive awareness for social acceptance among patients with substance use disorder. Further objectives are to analyze the relationships between the interesting variables as well as to explore the predictive role of craving to drugs with the emotional manipulation ability and the perceived acceptance.

### Design, setting, and sample

Research design: This study followed a descriptive correlational design.

### Setting

The study was carried out in the psychiatric outpatient clinic of Al-Maamoura Hospital for Psychiatric Medicine, which is affiliated to the Ministry of Health and Population. It serves three governorates, namely Alexandria, Behaira, and Matrouh. All people with mental illnesses and drug addictions receive free therapy at the outpatient clinic. This clinic operates six days a week, from 9 a.m. to 1 p.m.

### Participants

A convenience sample of 110 patients with substance use disorder from the sampling frame consisting of 400 patients (registered patients with substance use disorder who visited the outpatient clinic). The participants were estimated using G*Power Windows 3.1.9.7, software with the following parameters: power (1- β err probability) = 0.95, effect size = 0.5, α-error probability = 0.01, groups number = 1, and predictors = 3. The recruited participants were randomly using a random number generator program. Inclusion criteria are participants who are diagnosed by a psychiatrist to have substance use disorder with no co-morbidity according to DSM IV “as it is considered the manual used for the diagnosis of psychiatric and mental disorders follow the setting mentioned above.” Patients who were able to communicate coherently and relevantly were also included. Those who have drug-induced psychosis were excluded.

### Tools of the study

#### The Penn alcohol craving scale (PACS)

The Penn Alcohol Craving Scale (PACS) is developed by (Flannery, 1999) [15]. The PACS is a self-report questionnaire, a five-question that assesses the individuals’ desire for alcohol over the course of a week. The questionnaire asks questions about frequency, intensity, duration, and respondents’ capacity to refrain from drinking. The PACS shows strong internal consistency when utilized in numerous outpatient populations around the world [15]. The PACS values greater than 20 were regarded as meeting diagnostic criteria for craving for a diagnosis of substance use disorder. The PACS score has been explored as a stand-in for the craving criterion and was modified by Witkiewitz & Bowen (2010) to assess craving for all individuals with SUDs. It was also tested for validity, and internal consistency among patients with SUDs “Cronbach’s alpha was .87” [16].

#### Emotion manipulation questionnaire (EMQ)

The Emotional Manipulation Ability Scale [17] consists of 10 items derived from the emotional manipulation factor of Austin et al.’s (2007) measure [18]. It consists of three subscales; emotional manipulation, perceived poor emotional skills, and emotional concealment. Answers are given on a scale of 1 to 5, where one is for strongly disagreeing, and five is for strongly agreeing. High scores signify sophisticated emotional manipulation. The scale exhibits strong construct validity, and the internal consistency of the subscale is very good = 0.93 [5].

#### The perceived acceptance scale (PAS)

The Perceived Acceptance Scale was created to evaluate aspects of perceived acceptance that are relationship-specific [19] and was later modified by(González, Couñago, & MF, 2012) [20]. It is a 44-item self-report questionnaire created to examine how well people perceive their acceptance in four distinct types of relationships: friendships (12 items), family (12 items), mother (10 items), and father (10 items). Example items include “My parents objected to a number of things I did” or “I am a very important part of the lives of my friends.” Answers are made on a 5-point Likert-type scale ranging from 1 “strongly disagree” to 5 “strongly agree.” This scale has shown great convergent and discriminant validity and good internal consistency, with Cronbach’s alpha scores better than.86 for the subscale [20].

### Study procedure

#### Ethical considerations

The necessary formal approval and permission to conduct the study were obtained from the Research Ethics Committee of the Faculty of Nursing, Alexandria University; permission was taken from the Al-Maamoura Hospital, Ministry of Health in Egypt. The researchers reassured the patients about the anonymity and confidentiality of their responses and were informed about the purpose of the study. After that, an informed written consent was obtained. Also, they were told they had the right to refuse to participate in the study and that their decision would not affect their care. In addition, they were also told that they have the right to withdraw from the study at any time, even after starting, and that their privacy and confidentiality will be maintained.

#### Pilot study

After obtaining the official permissions from the responsible authorities, a pilot study was conducted on 11 patients with substance use disorder who met the inclusion criteria to evaluate the research instruments’ objectivity, transparency, viability, and applicability. The study sample did not contain the patients in this category. Cronbach alpha “non-parametric statistical test” was used to test tools’ internal consistency; at a level of significance (p ≤ 0.05). The Cronbach alpha reliability for Tool II(PACS), Tool III (EMQ), and Tool IV (PAS) reflected a high level of significance of “0.78”, “0.772, and 0.845, consequently. Tool I was developed by the researcher based on a review of relevant literature, and tool II, tool III, and tool IV were adopted. Five specialists in the field of psychiatric nursing and mental health revised the instruments to examine them for content validity, completeness, item clarity, and cultural relevance for Egyptian patients. The appropriate adjustments were made as a result.

### Data collection

The data collection was started by reviewing the recruited patients with substance use disorder’ medical charts using tool I to elicit their socio-demographic and clinical characteristics. A representative sample of patients with substance use disorder was recruited through the simple random method after excluding the patients who participated in the pilot study. Each participant was interviewed individually once by the researchers to collect the needed data using the study tools on the scheduled day for follow-up. Data were collected over a period of 4 months started.

### Data analysis

Data were coded and then fed to statistical software IBM SPSS version 20. Following data entry, checking and verification processes were carried out repeatedly to ensure that no mistakes were made during the data entry process. For clinical and demographic characteristics, descriptive statistics were used, and the numbers and percentages were used to describe and summarize qualitative data. Minimum and maximum were used for describing and summarizing quantitative data. Mean (X) was used to calculate the central tendency in statistical tests of significance. Standard deviation (SD) is an average of the deviations from the mean, and it was used to measure the degree of variability in a set of scores. The Kolmogorov-Smirnov test was used to check for normality in the distribution of quantitative variables. The Spearman coefficient was used to correlate between two distributed abnormally quantitative variables. All statistical tests were judged at a 0.05 significance level.

## Results

The study included 110 individuals with SUDs, with 90.9% of the respondents are male, those who aged between 18 and 35 years constituted 78.2%, and more than one-third were craft workers and earned diploma level (39.1%). Most of the participants are single (62.7%), more than half of the participants live in urban areas (57.3%), and 66.4% cohabitated with their families. Finally, most participants recorded that they did not have enough income (84.5%) (Table 1).

**Table 1:** Distribution of the studied cases according to demographic data (n = 110)

Demographic data	No.	%
Gender		
Male	100	90.9
Female	10	9.1
Age (years)		
18–35	86	78.2
> 35	24	21.8
Educational level		
Illiterate	19	17.3
Basic education	41	37.3
Secondary education or diploma	43	39.1
Higher education	7	6.4
Marital status		
Single	69	62.7
Married	41	37.3
Occupation		
No work	23	20.9
Student	11	10.0
Employee	13	11.8
Craft worker	43	39.1
Trade work	17	15.5
House wife	3	2.7
Residence		
Urban	63	57.3
Rural	47	42.7
Home participants		
Alone	8	7.3
With family	73	66.4
with relatives	4	3.6
With Husband/ wife	25	22.7
Financial status		
Not enough	93	84.5
Enough	14	12.7
More than enough	3	2.7
Source of income		
Work	71	64.5
Family	37	33.6
Subventions	2	1.8

Table 2 points out the descriptive analysis of the examined clinical variables. The analysis demonstrated that 77.3% of the participants began addiction at age ranged from 18 to 35 years old. More than half of the respondents, 62.7%, had 1 to less than five years of addiction. Also, the analysis of the main cause of substance addictions showed that the percentage of peer pressure, curiosity, and having access to drugs were obtained the highest percentage, 26.4%, 18.2%, and 14.5%, respectively. Addiction to opioids & CNS depressants are the most reported used substances, followed by Opioids& CNS depressants (19.1% and 15.5%, respectively).

**Table 2:** Distribution of the studied cases according to clinical data (n = 110)

Clinical data	No.	%
Age at the beginning of addiction		
< 18	15	13.6
18–35	85	77.3
> 35	10	9.1
Family history of addiction		
No	63	57.3
Yes	47	42.7
Duration of addiction (years)		
Less than 1	6	5.5
1 < 5	69	62.7
5 < 10	26	23.6
> 10	9	8.2
Previous hospitalization		
No	72	65.5
1 < 3 times	33	30.0
> 3 times	5	4.5
Number of substances		
1	33	30.0
2–4 substances	33	30.0
> 4 substances	44	40.0
Main cause of substance addictions		
Peer pressure	29	26.4
Aggressive behavior in childhood.	8	7.3
Neglect from parents or guardians.	11	10.0
Having access to drugs	16	14.5
Experimenting with drugs or other substances.	0	0.0
Curiosity	14	12.7
Psychological and family problems	20	18.2
Free time	12	10.9
Abused substances*		
Opioids “Tramadol- Heroin- Morphine-Codeine”	6	5.5
CNS Depressants “alcohol, barbiturates, anti-anxiety tranquilizers”	6	5.5
CNS Stimulants “cocaine, amphetamines, and methamphetamine”	6	5.5
Hallucinogens “Cannabis-LSD-MDMA-Ketamine- Pilocybin”	8	7.3
Inhalants” volatile solvents-Aerosol sprays-Gases-Nitrites”	3	2.7
Synthetic drugs	5	4.5
Opioids + Hallucinogens	17	15.5
Opioids + CNS depressants	21	19.1
Opioids + CNS Stimulants	13	11.8
CNS stimulants + Hallucinogens	11	10.0
Hallucinogens + Inhalants	8	7.3
CNS depressants + Hallucinogens	4	3.6
CNS stimulants + Inhalants	2	1.8

*More than one repsonse

**Fig. 1:**
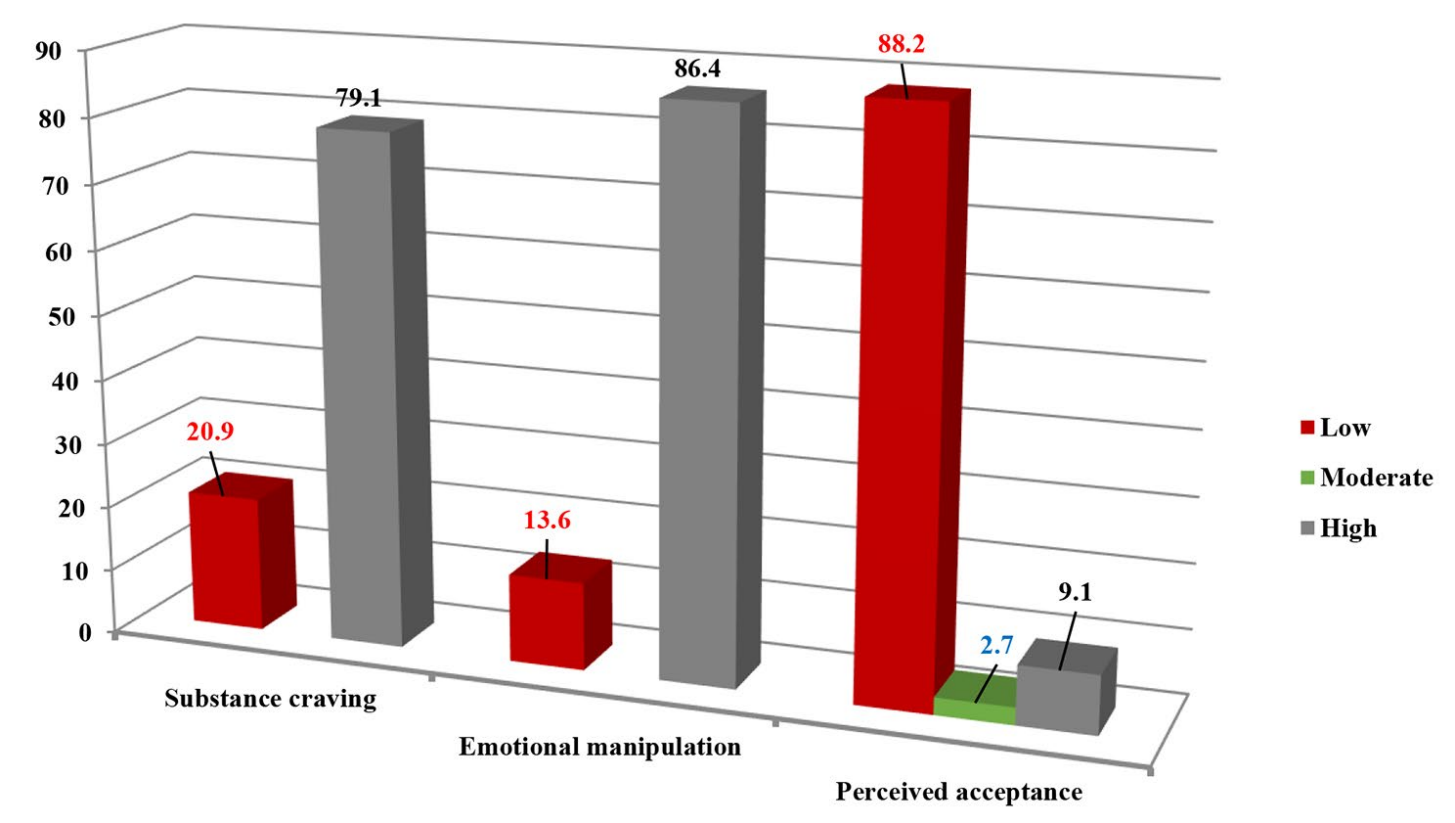
Levels of drug craving, emotional manipulation and perceived acceptance

As shown in Fig. 1, most of the respondents recorded high levels of PACS, and emotional manipulation ability (79.1%, 86.4%), respectively. On the contrary, the perceived acceptance scale demonstrated a low percentage (9.1%).

**Fig. 2:**
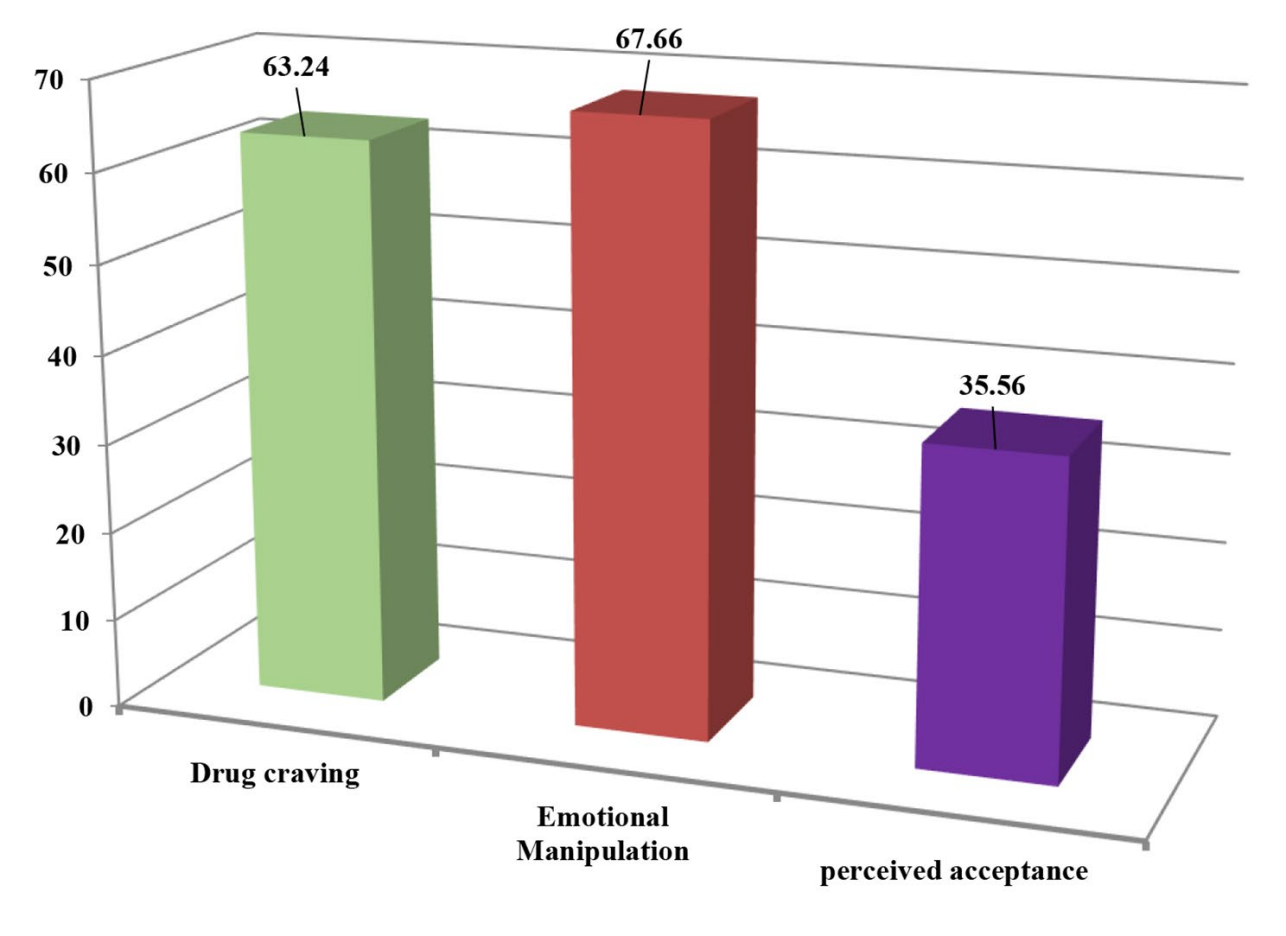
Total percentage mean score of PACS, emotional manipulation and perceived acceptance

The analysis of mean and standard deviation showed that the total percentage mean score of PACS, and emotional manipulation ability are relatively even at 63.24 ± 20.39 and 67.66 ± 21.0, respectively. However, the overall perceived acceptance scale demonstrated the lowest mean score of 35.56 ± 16.13, Fig. 2.

**Fig. 3:**
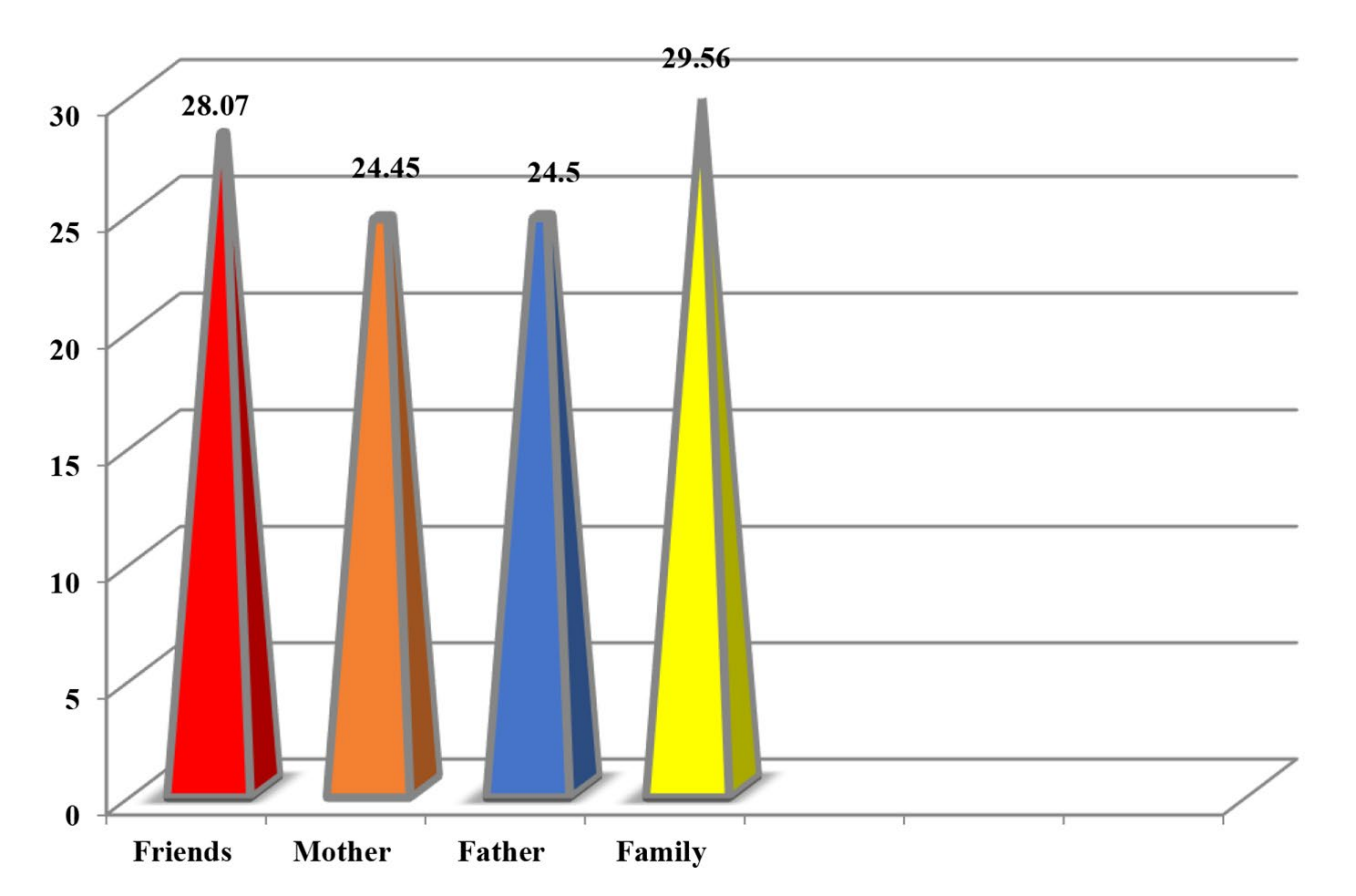
Mean scores of perceived acceptance

Figure 3. demonstrates the mean score of acceptance as perceived by individuals with substance use disorders. The highest mean score of perceived acceptance was received from participants’ family and friends (29.56 ± 8.23 and 28.07 ± 8.55, respectively), followed by father and mother are almost the same (24.50 ± 6.77 and 24.45 ± 6.41, respectively).

Results of the Pearson’s correlation shown in Table 3 indicates that the scores on emotional manipulation ability and PACS were highly and positively correlated (r = 0.374; p < 0.001). This indicates that the higher the level of emotional manipulation ability, the higher the level of craving for drug use. However, an inverse relationship of the perceived acceptance scale with PACS (r = 0-0.234; p < 0.01).

**Table 3:** Correlation between Penn Alcohol Craving Scale (PACS), Emotional manipulation ability and the perceived acceptance scale (n = 110)

	Penn Alcohol Craving Scale (PACS)	
	**r**_**s**_	**p**
Emotional manipulation ability	0.374^*^	< 0.001^*^
The perceived acceptance scale	-0.234^*^	0.014^*^

r_s_: Spearman coefficient *: Statistically significant at p ≤ 0.05

Table 4. After determining the significant correlations, a multivariate linear regression analysis was performed to determine if PACS value was predictive of emotional manipulation ability and the perceived acceptance. As the table illustrates, PACS was a significant predictor of the emotional manipulation ability (β = 0.581, p = 0.001). This indicates that the higher the PACS, the higher the emotional manipulation ability among individuals with substance use disorders. However, the PACS was no longer significantly associated with perceived acceptance (β = 0.088, p = 0.930). The overall model fit was R^2^ = 0.331 (F = 26.498, p = 0.001).

**Table 4:** Multivariate analysis linear regression for Penn Alcohol Craving Scale (PACS)

	B	Beta	t	p	95% CI	
					**LL**	**UL**
Emotional manipulation ability	0.565	0.581	5.579^*^	< 0.001^*^	0.364	0.765
The perceived acceptance scale	0.012	0.009	0.088	0.930	-0.250	0.273
R^2^ = 0.331,F = 26.498^*^,p < 0.001^*^						

F,p: f and p values for the model R^2^: Coefficient of determination B: Unstandardized Coefficients

Beta: Standardized Coefficients t: t-test of significance CI: Confidence interval

LL: Lower limit UL: Upper Limit *: Statistically significant at p ≤ 0.05

## Discussion

Cravings, the main feature of addiction, are the drivers of continued drug abuse and a return to addiction after recovery [21]. It is deemed a substantial factor contributing to the risk of “quitting failure” and behavioral conduct problems as well [22]. Therefore, this study would ultimately act as an impetus for identifying the relationship between drug cravings, emotional manipulation, and introspective awareness among patients with substance use disorder.

The current study revealed that most patients with substance use disorders demonstrated a high level of craving. This finding supports testimonials registered in numerous studies [23–26]. One explanation for this finding might be grounded in the socio-demographic and clinical data characteristics of the participants. For instance, a high proportion of the respondents were aged between 18 and 35 years. Sharma et al. (2018) pointed out that this age period is commonly characterized by exposure to a wide array of daily life hassles. Including but not limited to peer pressure, curiosity to try out offered substances, juggling responsibilities such as looking for a career, and being financially independent [27]. The stressors that might trigger their tendency to abuse substances as a resort of coping and lessen the impact of emotional pain that has arisen from the encountered stressors [28].

In the same vein, more than three-quarters of the respondents were handicraft workers. This corresponds with the findings of other studies by Muller et al. (2019), who testified that fewer than three fifths of the studied subjects were handicraft workers [29]. Ibrahim et al. (2022) reported that less than half were handicraft workers [26]. In fact, this work area has easy acceptability and feasibility of substances. Besides, the misconception and conviction that could prevail among Egyptian people that substances improve their physical and sexual abilities [30].

Scientific studies argue that drug addiction is an illness marked by self-delusion, denial, mystification, and dishonesty. Emotional manipulation is frequently seen as a prevalent behavioral pattern among patients with substance use disorder [7, 31]. Caputo (2019) suggested that the urge to get substances should allow patients with substance use disorder to use deception to influence or control others. They are likelier to make fake promises, act the victim, invent groundless justifications for carelessness make people feel uncomfortable or guilty to accommodate excessive expectations, and threaten self-harm [32]. All of these would probably lend further support to the existing findings, in which a large majority of the participants recorded a high level of emotional manipulation and, consequently, almost all of them perceived poor social acceptance from their parents, families, and friends. In a study conducted by Rachel et al. (2019), a high level of social rejection was demonstrated by patients with substance use disorder, and they attributed their findings to the burden imposed on the patients’ families and friends [33].

Pearson’s correlation coefficient displayed that craving for drugs was significantly and positively correlated with emotional manipulation and perceived social acceptance. This finding dovetails with the reports of Ferrari et al. (2008) and Sher & Epler (2004), who attested that lying and dishonesty are relatively common reported behaviors among patients with substance use disorder [31, 34]. They are frequently more inclined to manipulate those around them. In this context, prior studies have established a link between drug use disorders, psychopathic personality features, and more antisocial and deviant relationship behaviors [35, 36]. However, our work didn’t precisely measure this. This mandates the need to explore and examine the extent to which craving for drugs could affect personality structure.

Researchers in the current study noted that craving for drugs didn’t predict perceived social acceptance. However, this result was surprising because the investigators expected the opposite trend. This looks paradoxical in that researchers hypothesize that the higher the level of craving, the higher the level of perceived social acceptance. A reasonable explanation for this finding is that patients with substance use disorder usually struggle to defend themselves from a sense of shame, guilt, or remorse [37]. They deny their problem and escape from facing the harsh reality. From Freud’s perspective, denial is a defense mechanism commonly used by substance abusers [38]. In this scenario, patients with substance use disorders have clouded insight into their problems. This puts them at high risk of rejection by their loved ones and those with whom they interact.

Further loss of social ties, bonding with others, and being continually exposed to an exaggerated dose of negative criticism from significant others [39]. Relatedly, Leventhal et al. (2011) signified that social acceptance problems increase the risk of substance use [40]. Among adults, social rejection is associated with drug consumption, and loneliness is associated with a craving for drug use [37]. Laws et al. (2017) vowed that individuals usually cope with feelings of social rejection by drinking more than usual to reduce stress. Moreover, social rejection may lead individuals to seek greater social acceptance and the exhilaration experienced in social drinking [41].

### Limitations

The researchers would like to remark on some limitations of the current study. First, we relied on the respondents’ self-reports of the standardized measurement tools; thus, biases in the reporting of measures may affect the existing findings. Another limitation is the relatively small sample size, which could limit our ability to generalize the obtained data. The causes of drug-related hospital admissions are varied. It might be voluntary treatment applications, under probation, or compulsory hospitalizations [42, 43]; however, this aspect wasn’t included in the clinical characteristics of the studied patients. Finally, our work only tested the correlation between the interesting variables, so we did not have enough social interaction data in the current study to test the hypothesis that craving led to reduced interoceptive awareness of social acceptance. Future research will benefit if we investigate the relationship between the interesting variables in both directions.

## Conclusions

It can be concluded from the study that craving for drugs was significantly and positively correlated with emotional manipulation and perceived social acceptance. Craving for drugs was a significant predictor of emotional manipulation ability. Incorporation of effective nursing interventions to enable patients with substance use disorder to engage in self-reflection related to how their cravings for drugs may lead them to prioritize their needs over others.

## Data Availability

The datasets used or analyzed in this study are available from the corresponding author upon request.

## Abbreviations

DA: Drug addiction
SUDs: Substance Use Disorders
DSM: Diagnostic and Statistical Manual of Mental Disorders
WHO: World Health Organization
PACS: Penn Alcohol Craving Scale
EMQ: Emotion Manipulation Questionnaire
PAS: The Perceived Acceptance Scale
